# Engineering Azeotropy to Optimize the Self-Assembly of Colloidal Mixtures

**DOI:** 10.1021/acsnano.3c05569

**Published:** 2023-12-04

**Authors:** Camilla Beneduce, Francesco Sciortino, Petr Šulc, John Russo

**Affiliations:** † Dipartimento di Fisica, Sapienza Università di Roma, P.le Aldo Moro 5, 00185 Rome, Italy; ‡ School of Molecular Sciences and Center for Molecular Design and Biomimetics, The Biodesign Institute, Arizona State University, 1001 South McAllister Avenue, Tempe, Arizona 85281, United States; § School of Natural Sciences, Department of Bioscience, TU Munich, Am Coulombwall 4a, 85748, Garching, Germany

**Keywords:** azeotropy, self-assembly, multicomponent mixtures, nucleation, patchy particles, DNA origami

## Abstract

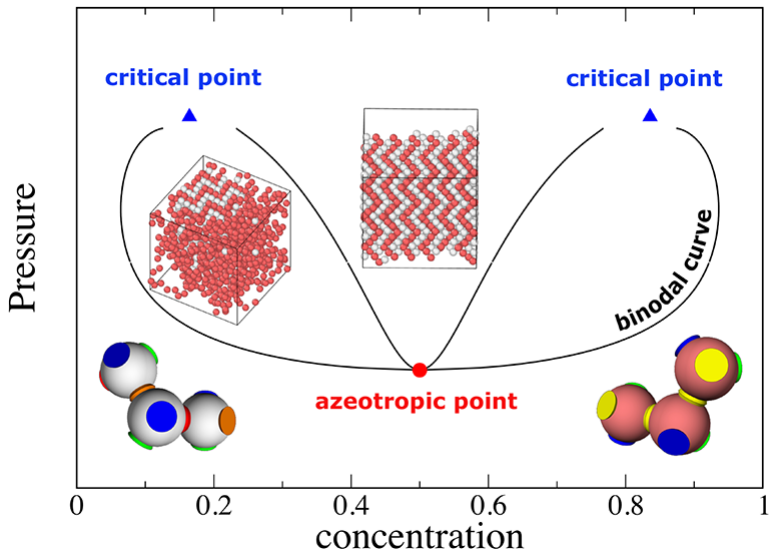

The goal of inverse self-assembly is to design interparticle interactions capable of assembling the units into a desired target structure. The effective assembly of complex structures often requires the use of multiple components, each new component increasing the thermodynamic degrees of freedom and, hence, the complexity of the self-assembly pathway. In this work we explore the possibility to use azeotropy, i.e., a special thermodynamic condition where the system behaves effectively as a one-component system, as a way to control the self-assembly of an arbitrary number of components. Exploiting the mass-balance equations, we show how to select patchy particle systems that exhibit azeotropic points along the desired self-assembly pathway. As an example we map the phase diagram of a binary mixture that, by design, fully assembles into cubic (and only cubic) diamond crystal via an azeotropic point. The ability to explicitly include azeotropic points in artificial designs reveals effective pathways for the self-assembly of complex structures.

When interactions between particles in a dilute fluid phase have strength comparable to or larger than the thermal energy, the fluid becomes unstable and the particles condense searching for a lower free energy state. The spontaneous formation of interparticle bonds gives rise to aggregates whose final state can be either that of an ordered lattice, a connected percolating structure (e.g., a liquid), or a collection of finite size clusters. When finite size or periodic structures are formed, this spontaneous search for the lowest free energy state is called *self-assembly*.^1,2^

While the computation of the free energy of a structure is a laborious but solved problem in statistical mechanics, several challenges hamper our understanding of self-assembly and our ability to mimic natural systems. In the *direct* self-assembly problem, one starts from a set of predetermined elementary units with known interparticle interactions and is tasked with selecting structures that correspond to free energy minima. This is done either with intuition (for simple structures), with brute force approaches (direct molecular simulations), or with specialized algorithms.^3,4^ Even more challenging is the *inverse* self-assembly problem, where one is tasked with designing the interparticle interactions that will self-assemble a desired target structure.^5,6^ In this case the problems are 2-fold: first designing an interaction-potential, second confirming that there are no alternative structures that preempt the formation of the target one.^7^ So far, two types of approaches have been explored: optimization algorithms and geometrical strategies. Optimization algorithms allow one to design a pair potential whose free-energy minima is guaranteed to be the desired structure.^6,8−16^ However, the interparticle interactions that result from such procedures are often too complex and require a degree of precision that is out of reach for experimental realization. In geometrical strategies, instead, one matches the geometric features of the target structure by tuning some interaction properties of the building units, e.g., the shape and the directionality of the bonds, in order to match the geometric features of the target structure.^17−24^ Although it is an experimentally feasible approach, it is system specific, and it requires a high degree of geometrical intuition.

A different solution strategy to the inverse self-assembly problem is to extend the number of building blocks, going from single component systems to multicomponent mixtures, shifting the problem of designing complex single particle potentials to that of optimizing simpler (and more geometrical) interactions between multiple components.^7,25−30^ Extending the *alphabet* of building blocks, i.e., the number of components, lowers the degree of symmetry in the final structure, allowing for a considerable reduction in competing structures, and an easier assembly pathway toward the target design. Compared to single-component mixtures, and leaving experimental challenges aside, two major problems are introduced by the increase in the number of components: a combinatorial problem and a thermodynamic problem.

The *combinatorial problem* arises from the fact that each new component increases exponentially the space of possible solutions and with that the computational time required to find a solution. To tackle it, advanced optimization algorithms are necessary, such as genetic algorithms^31^ or machine learning techniques.^16,32^ Some of us have recently introduced an approach called *SAT-assembly*,^26,33^ which encodes the bond topology of the target structure into a system of Boolean equations (a satisfiability problem commonly named SAT) whose solution gives the interaction matrix between different patches. The sophistication of modern SAT solvers^34^ allows to effectively tackle the combinatorial problem for complex assemblies, including open crystalline structures, photonic crystals, and clathrate structures.

The *thermodynamic problem* arises instead because, according to Gibbs rule of phases,^35^ each component represents an additional thermodynamic degree of freedom of the system, extending the phase behavior phenomenology in ways that can interfere with the self-assembly pathway. No general strategy to tackle this problem has so far been proposed. Full phase diagram calculations are in fact very time-consuming and are often avoided in multicomponent systems due to their complexity. The goal of this article is to show how to overcome the thermodynamic difficulties associated with the use of multicomponent mixtures by explicitly encoding azeotropic points in the self-assembly designs of patchy particles. The azeotropic point is a point where the free-energy of the mixture can be written as that of an effective one-component system (see Supporting Information I for a concise explanation of azeotropy), a condition that ensures that coexisting phases will have the same concentration as the parent homogeneous system. The ability to explicitly include azeotropic points along the self-assembly pathways of these systems represents an attractive strategy to tame the complexity in phase behavior usually associated with multicomponent mixtures. Some of the advantages of combining azeotropic behavior with self-assembly are listed here. (i) The ability to (considerably) increase the reaction rates of the self-assembly process by quenching the system in a region of (liquid–gas) metastability: in fact, it is well-established that for one-component systems nucleation rates increase in proximity of density fluctuations like the ones found near liquid–gas critical points^36^ and spinodal loci.^37^ (ii) Increase the kinetics of the self-assembly reaction: if the concentration of the azeotropic point is the same as the crystal composition, one can avoid slow diffusion-limited processes, where the crystal nucleus has to wait for the concentration of the local environment to match the one of the target structure.^38^ (iii) The yield of the self-assembly process can proceed theoretically until all components are exhausted (to 100%), as the liquid phase will form at the same composition of the target crystalline structure.

In this article, we will first show that it is indeed possible to effectively control the self-assembly of suitably designed patchy particles by exploiting the encoded azeotropic properties. As a proof of concept, we then investigate in detail a binary mixture that is designed to form (only) the cubic diamond crystal. This mixture also shows a very interesting phase behavior, where phase-separation only occurs for mixed states and not for the pure components.

## Results and Discussion

**Figure 1:**
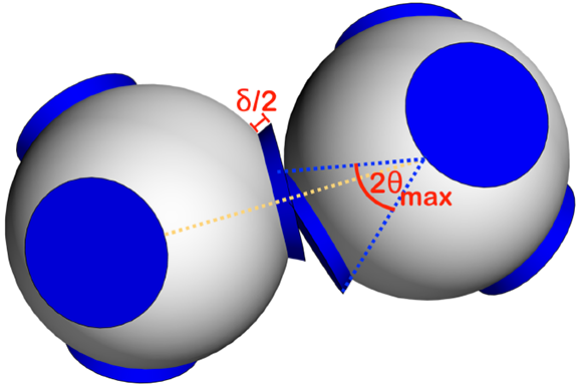
Patchy particles schematic. Two patchy particles with four tetrahedrally arranged patches (in blue) interacting with the Kern–Frenkel potential defined in the Patchy Particles section in the Methods.

Our results pertain to systems whose components aggregate by forming bonds, i.e., to the vast class of associating systems.^39^ The main assumption is that the systems are in equilibrium and that bond formation is controlled by a mass-balance equation. We propose general design rules that realize azeotropy in any system that satisfies these conditions. To demonstrate the effectiveness of our approach we will give concrete examples that considers mixtures of patchy particles (Figure 1). For example, patchy particles can be realized with DNA origami, engineering distinct regions on the origami structures with complementary DNA sequences that precisely match the complementary sequences on the functionalized nanoparticles. This design enables selective binding and controlled assembly through hybridization interactions, leading to the formation of patchy particles with specific binding sites. A detailed description of the connections between DNA-origami and patchy particles is reported in the Methods section. For these mixtures of patchy particles, thermodynamic properties will be computed both via Wertheim’s first order perturbation theory,^39−48^ and via molecular simulations, both confirming the presence of the azeotropic point embedded in the phase diagram by design. In particular, to calculate the phase behavior of the studied systems theoretically, we adopt the isochoric thermodynamic’s framework, while to study it numerically, we implement Monte Carlo simulations in the Gibbs ensemble. All these techniques are summarized in the Methods section.

In the rest of this article energy is measured in units of the square-well depth (ϵ), distances in units of the patchy particle diameter (σ), pressure in units of ϵ/σ^3^ and *k*_B_ = 1.

### Law of Mass Action

In deriving the azeotropy conditions we will make use of the law of mass action,^41,44,47^ which quantifies the probability for a patch α to be nonbonded, and which we denote by *X*_α_^(*i*)^, where the index α runs over all patches of species *i*
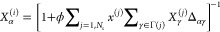
1where Γ(*j*) is the set of all patches of species *j*, Δ_αγ_ quantifies the strength of the interaction between patches α and γ, and ϕ is the total packing fraction. A detailed expression for Δ_αγ_ is reported in the Methods section, but in the remainder we will consider the following simplification: any pair of interacting patches forms bonds of the same type (bonding volume *V*_*b*_ and energy ϵ), so that Δ_αγ_ = Δ if α and γ interact, or Δ_αγ_ = 0 if they do not. We call **ϒ** the *interaction matrix*, whose elements ϒ_αγ_ = Δ_αγ_/Δ are ones if patches α and γ interact, and zeros if they do not. By construction, **ϒ** is a symmetric matrix (if patch α binds with patch γ, then also patch γ binds with patch α).

One possible strategy to compute **ϒ**, i.e., to determine which pair of patches should interact, such that the particles will self-assemble into a desired structure, is the *SAT-assembly* framework.^26^ Here we focus on the general conditions one needs to impose on ϒ_αγ_ in order to obtain azeotropic mixtures, regardless of the desired target structure.

### Azeotropy Design Rules

We consider a *N*_*s*_-component mixture with all species having the same diameter σ, the same number (*N*_*p*_) and placements of patches, and differing only in the patches type (patches color). We first notice that a sufficient condition for azeotropy is obtained by imposing that all probabilities *X*_α_^(*i*)^ in eq 1 are the same for all patches in the system, *X*_α_^(*i*)^ = *X*. In this way, all species will behave like an effective one-component system where all bonds have the same probability to be formed. The same condition can be demonstrated to hold within Wertheim’s perturbation theory: in the Wertheim Perturbation Theory section in the Methods we notice that the equality of all *X*_α_^(*i*)^ implies that the Helmholtz bonding free energy (eq 15) reduces to that of a one-component system.

In order to determine whether there is a thermodynamic point where all *X*_α_^(*i*)^ have the same value, we turn to the mass balance condition, eq 1, which is a set of *N*_*s*_ × *N*_*p*_ equations in the variables *X*_α_^(*i*)^. Looking for the rules under which all the mass balance equations become equivalent provides a sufficient condition for the appearance of azeotropy in a multicomponent mixture.

In the following, we examine three families of rules that ensure azeotropy:

• The *bond exclusivity* condition. This rule generates azeotropic points at equimolar conditions.
• The *bond multiplicity* condition. This rule allows for azeotropic points at nonequimolar conditions.
• The *fully connected bond* condition. This rule generates always-azeotropic mixtures, e.g., where the concentration remains the same during demixing for every point in the coexistence region.

#### Bond Exclusivity Condition

One condition ensuring azeotropy is the *bond exclusivity* constraint requiring that each patch has only one bonding partner (that can be itself in the case of self-complementarity) among all patches of all species in the mixture. This implies that all patches are different and that **ϒ** has a single one for each row, located at a different column for different rows. This condition, with its symmetric bonding rules, can be realized when two species of particles are functionalized with complementary DNA strands, a system which has found great success in nanotechnology.^49,50^

We consider here the case where all bonds have the same bonding energy such that azeotropy appears at equimolar conditions: a *N*_*s*_-component mixture will be azeotropic if it is prepared by mixing all of the *N*_*s*_ components at the equimolar concentration 1/*N*_*s*_. To see this, we note that the bond exclusivity condition implies that the sum over the patches (*∑*_γ∈Γ(*j*)_) and the sum over the species (∑_*j*=1,*N*_*s*__) in eq 1 reduce to a single contribution since patch α belonging to species *i* can interact only with its partner patch γ belonging to species *j* (*j* can be also equal to *i* as well as α can be equal to γ). Therefore, the *N*_*s*_ × *N*_*p*_ mass balance equations for *X*_α_^(*i*)^ reduce all to equations of the form
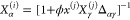
2which couple only *X*_α_^(*i*)^ with *X*_γ_^(*j*)^. Moreover, by designing bonds with the same strength, Δ_αγ_ ≡ Δ for all patches α and γ. By considering the pair of equations for *X*_α_^(*i*)^ and *X*_γ_^(*j*)^ one obtains, without knowing the exact patchy particles design, that the *N*_*s*_ × *N*_*p*_ mass balance equations become all equivalent to
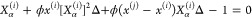
3

With the equimolarity condition, *x*^(*i*)^ = 1/*N*_*s*_, the *N*_*s*_ × *N*_*p*_ equations above admit the azeotropic solution *X*_α_^(*i*)^ = *X*, where *X* is the solution of
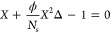
4

Thus, the bond exclusivity condition generates an azeotrope at equimolar concentration, which can be exploited to self-assemble target structures composed of an equal number of all species. An example of interaction matrix satisfying the bond exclusivity condition is given in the next section, where we will verify explicitly the presence of an equimolar azeotropic point not only with Wertheim’s thermodynamic theory but also explicitly with Monte Carlo simulation of a patchy particle realization of the interaction matrix.

The bond exclusivity condition is easily generalized to cases where multiple-bonding is allowed (one patch capable of bonding to more than one patch), a case that can be realized with DNA functionalization, as explained in Supporting Information IV and/or when the patches are not distinct (when the interaction matrix has repeated columns or rows, i.e., when its determinant is zero). In these cases, to have equimolar azeotropy conditions, one needs to ensure that every patch has the same total number (*m*) of bonding partners (distributed over one or more species). In this case the mass-balance equation admits the solution *X*_α_^(*i*)^ = *X* (azeotropy), with *X* satisfying the following equation
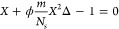
5

#### Bond Multiplicity Condition

A simple generalization of the bond exclusivity condition allows moving the azeotropic point to off-equimolar conditions. Considering a binary mixture where the ratio between the two species (denoted as (1) and (2)) is 1:*n*, in order to have an azeotrope at *x*^(2)^ = *nx*^(1)^ (i.e., *x*^(1)^ = 1/(*n* + 1) and *x*^(2)^ = *n*/(*n* + 1)) it is sufficient to enforce

• bond exclusivity to all patches bonding to species (2), i.e., each patch has a unique bonding partner with species (2).
• *n*-bond multiplicity to all patches bonding to species (1), i.e., each patch has *n* bonding partners with species (1).

With these conditions, all mass balance equations, eq 1, admit the azeotropic solution *X*_α_^(*i*)^ = *X* with *X* satisfying the following equation
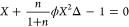
6

The *bond multiplicity* rule is a generalization of the previous bond exclusivity case, that we recover if *n* = 1. This recipe is generalizable to multicomponent mixtures with more than two species: the *bond multiplicity* condition will require to establish a bond with *m* patches belonging to certain species, where *m* is the least common multiple between component ratios.

An explicit example of a binary system of patchy particles with bond multiplicity is reported in Supporting Information II, and an example of a ternary system having an azeotropic point off equimolar conditions is reported in Supporing Information IV.

In short, bond multiplicity provides a way to shift the azeotropic point at a concentration different from the equimolar one. However, we underline that, with the presented rules, once the number of species and of patches is set, it is not possible to design a mixture exhibiting azeotropy at arbitrary concentration. For instance, for a binary mixture with four patches tetrahedrally arranged, there is no design satisfying our bonding rules for the ratio 1:3. More general conditions can be built by lifting the requirement that all bonds have the same energy, Δ_αγ_ ≠ Δ, but bearing in mind that a fine control over bonding energies represents a significant experimental challenge.

#### Fully Connected Bond Condition

The *fully connected bond* condition introduces bonding rules that ensure full azeotropy at all concentrations without the need to tune bonding energies. In this case, the concentration of the two coexisting phases is always constant during demixing. For a general *N*_*s*_-component mixture of patchy particles with *N*_*p*_ patches, the *fully connected bond* condition is achieved when each patch can bind with *N*_*s*_ patches, each located on a different species. In this case the sum *∑*_γ∈Γ(*j*)_ in the mass balance equation (eq 1) drops out, as there is only one bonding partner on each species, becoming
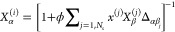
7where the patch β on particle *j* is the unique bonding partner of patch α on species *i*. Now, assuming that all bonds are of the same type, Δ_αγ_ = Δ, and remembering that *∑*_*j*_*x*^(*j*)^ = 1, we see that the mass balance equation admits azeotropic solutions *X*_α_^(*i*)^ = *X* where *X* satisfies
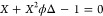
8

A possible interaction matrix for a binary mixture satisfying the fully connected bond condition is reported in Supporting Information III and a DNA implementation in Supporting Information V.

### Application to Cubic Diamond Crystals

**Figure 2:**
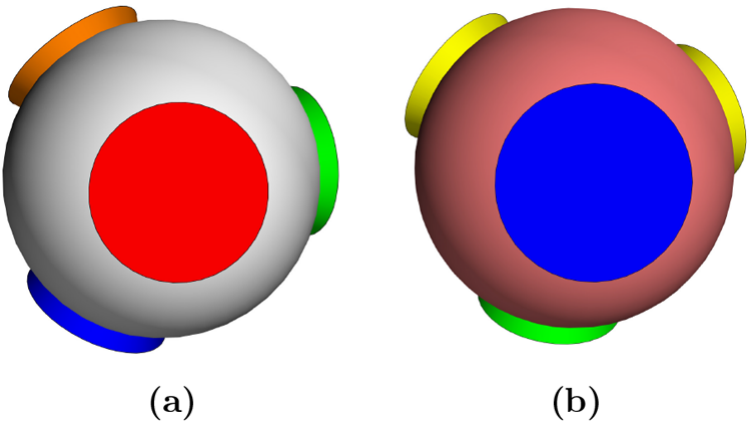
3D representation of the two patchy particles species (a) and (b) of the SAT-designed N2c8s2 binary mixture. Equal patch colors indicate which patches can bind to each other and the colors appearing only once are assigned to self-interacting patches.

One of the most interesting and challenging bottom-up realizations of a target structure is that of the cubic diamond.^51,52^ Realizing a cubic diamond on colloidal scale would allow the creation of a photonic crystal that allows for light manipulation in a controlled way.^53−55^ The self-assembly of a cubic diamond is complex since its lattice is an open structure which competes with the hexagonal diamond structure, which prevents the cubic diamond from forming without defects such as stacking faults.^56^ Several studies have been performed to overcome these difficulties,^56,57^ including solutions obtained within the SAT-assembly framework.^26,33^ Because of the topology of the cubic diamond lattice, patchy particles of valence four with a tetrahedral arrangement of the patches are used to self-assemble the crystal. The minimal SAT-designed solution (the one requiring the smallest number of distinct particles) is the so-called N2c8s2 binary mixture^58^ that uses two species (N2), eight patches types (colors) (c8) and two self-interacting colors (s2) and it is schematized in Figure 2 where colors identify the interacting (and not the different) patches according to the interaction matrix **ϒ**. Note that the number of self-interacting colors is also the trace of matrix **ϒ**.

The N2c8s2 interaction matrix, encoding the design with 2 species and 8 distinct patches (or colors), is
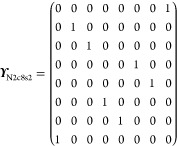
9

We notice that having a single one for each row, the bond exclusivity condition is satisfied, and we thus expect to find an azeotrope line at concentration *x*^(1)^ = *x*^(2)^ = 1/2. The N2c8s2 mixture is thus an ideal candidate to test the appearance of azeotropy and to investigate in detail its self-assembly properties.

In order to verify the effective presence of an azeotrope when the two species are mixed at equal ratio, we first use Wertheim’s theory^40^ to determine the binodal curve in pressure–concentration and density-concentration phase diagrams. The thermodynamic conditions for a stable state of the mixture at constant pressure and temperature are found when the Gibbs free energy per particle *g* has a minimum. *g*, the Legendre transform of the Helmholtz free energy per particle *f*, is defined as
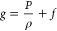
10where *P* is the pressure and ρ is the total number density. Since the same total density can be achieved by mixing species at more than one pair of concentrations *x*_1_ ≡ *x* and *x*_2_ = 1 – *x*_1_, first we must minimize *g* for each fixed concentration *x* with respect to the density ρ. In this way, the Gibbs free energy becomes only a function of concentration. Coexisting phases having the same temperature, pressure and chemical potential can be obtained by searching those points on *g*(*x*) that are connected by a common tangent.^42^ Starting from a single pair of coexistence points found with the common tangent rule, we use the isochoric thermodynamics equations (Isochoric Thermodynamics section in the Methods) to trace the coexistence lines as a function of concentration and pressure. For all the following numerical calculations, we fix the potential parameters to the values cos θ_max_ = 0.98 and δ = 0.2. This choice follows from previous studies demonstrating that the nucleation is facilitated at small apertures of the angle θ_max_.^39,56,59^

**Figure 3:**
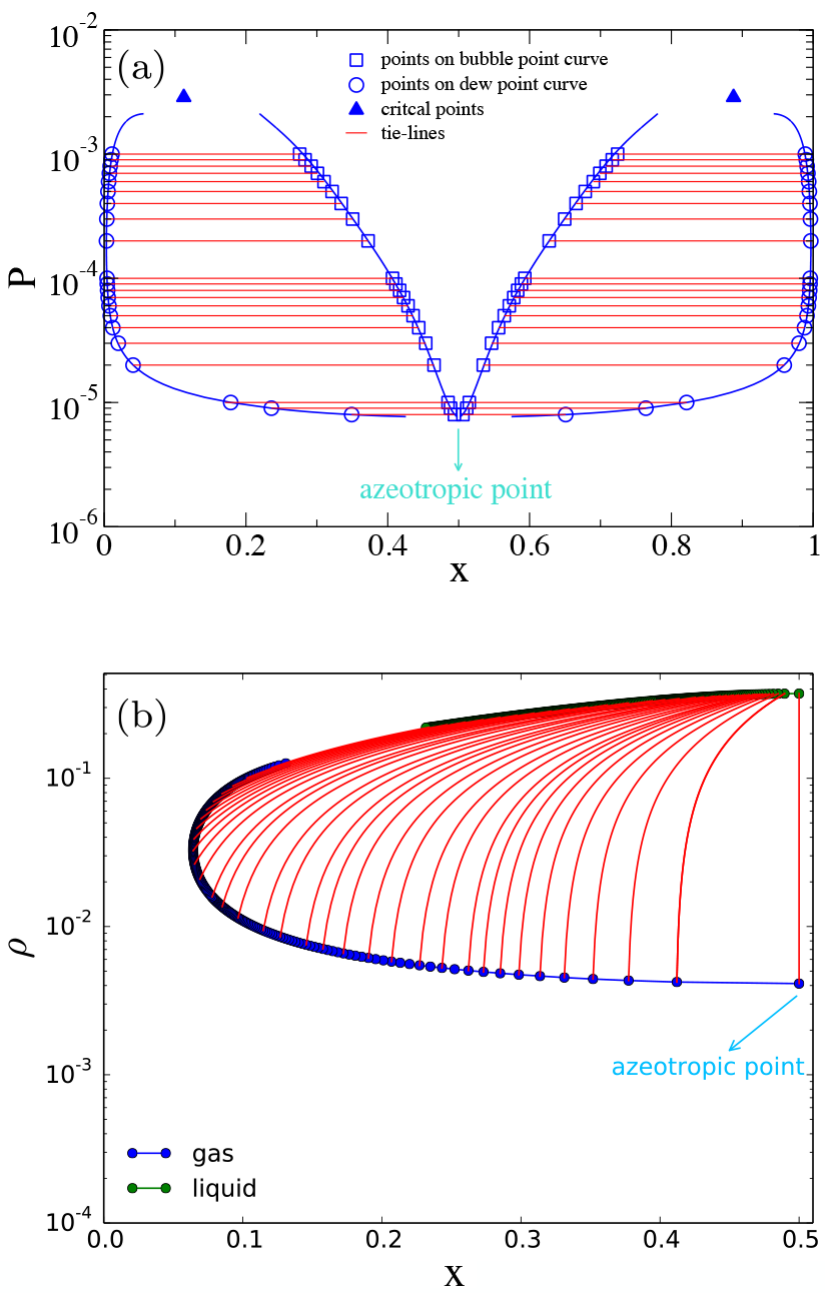
Wertheim pressure-concentration (a) and density-concentration (b) phase diagrams for the N2c8s2 SAT-designed binary mixture. The (a) phase diagram is computed at temperature *T* = 0.07 while the (b) one at temperature *T* = 0.08, *x* is the concentration of the first species. In (a) circles and squares, connected by red tie-lines, represent the coexistence points obtained from the common tangent construction on the Gibbs free energy curve. Blue lines indicate the binodal curve computed by numerically integrating eq 24. Triangles are at the location of binary critical points. In (b) the only vertical tie-line is the one at the azeotropic concentration: only a binary mixture prepared in a homogeneous phase at the azeotropic concentration retains the original ratio between components when it phase separates. Tie-lines are not straight since the density axis is in logarithmic scale.

The pressure concentration phase diagram obtained at *T* = 0.07 is shown in Figure 3a. This phase diagram confirms that the N2c8s2 has an azeotropic point at concentration equal to *x* = 0.5: it is exactly at *x* = 0.5 that the bubble point curve (where the vapor phase first appears when pressure is lowered starting from a point greater than the total vapor pressure^60^) and the dew point curve (where the liquid phase first originates when pressure in increased starting from a point in the vapor phase^60^) are tangent and the coexistence region reduces to a point. Moreover since the azeotrope is at the lower extremum in the pressure–concentration phase diagram, the N2c8s2 binary mixture is a negative azeotropic binary mixture.^60,61^

In Figure 3b we plot the coexistence region in the density-concentration phase diagram. The azeotropic nature of the solution with *x* = 0.5 is evident from the slope of the tie-lines: only at *x* = 0.5 is the tie-line a vertical line, indicating that only if the binary mixture is prepared by mixing together an equal concentration of the two species will the coexisting phases preserve the same concentration.

**Figure 4:**
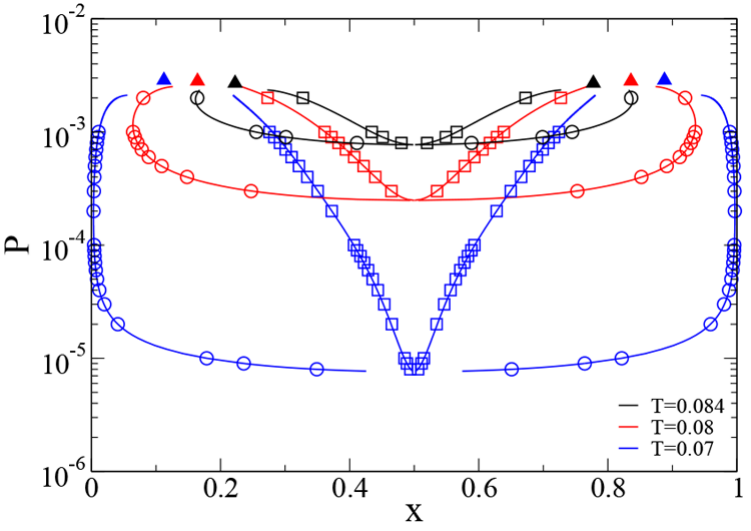
Comparison of Wertheim pressure-concentration phase diagrams for the N2c8s2 SAT-designed binary mixture at temperatures *T* = 0.07, *T* = 0.08, and *T* = 0.084. Circles represent points belonging to the dew point curve. Squares represent points belonging to the bubble point curve. Triangles indicate critical points.

Unexpectedly, the shape of the coexistence regions in the *P* – *T* plane (sometime called the phase diagram “topology”^60,61^) indicates that the N2c8s2 mixture belongs to a distinctive type of binary phase diagram in which the pure components (*x* = 0 and *x* = 1) do not have a liquid–gas transition but their mixture does. Figure 4 shows that with decreasing temperature, the coexistence region becomes larger without ever crossing the limit concentrations *x* = 0 and *x* = 1. The topology of the phase diagram is equivalent to that of an ordinary azeotropic binary mixture but in which the binary critical point line goes to (*P*, *T*) → 0 as the concentration goes to *x* → 0 or *x* → 1.

**Figure 5:**
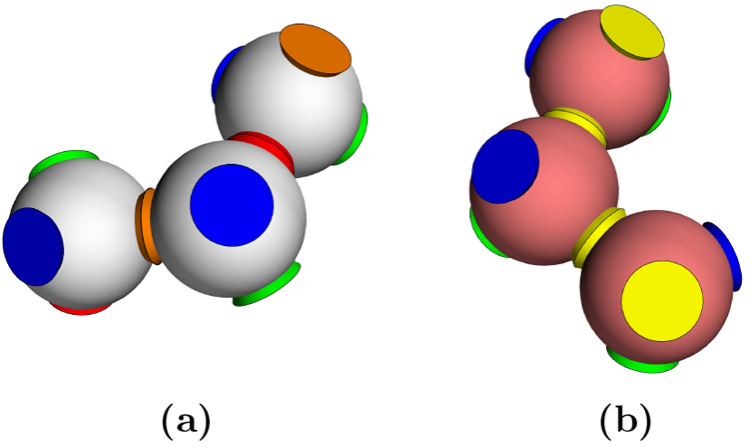
In single component systems only chain aggregates can form. If the SAT-designed N2c8s2 binary mixture becomes a single component system, composed either just by patchy particles of the first species (a) or just by patchy particles of the second species (b), patchy particles can aggregate only forming chains, i.e., they behave like patchy particles with valence two.

This unconventional behavior is originated by the fact that patchy particles of the same species can bind to each other with no more than two bonds, as encoded in the interaction matrix (eq 9). Hence, under pure component conditions, particles can aggregate only into chains as depicted in Figure 5. Therefore, even if particles have four patches, when *x* = 0 or *x* = 1 they behave like bifunctional particles and hence have no liquid–gas phase separation.^43^ We note that the idea that systems with two-patches have a hidden critical point at *P* = 0 and *T* = 0 has been recently revisited in ref (62) and generalized to colored patches in ref (63).

**Figure 6:**
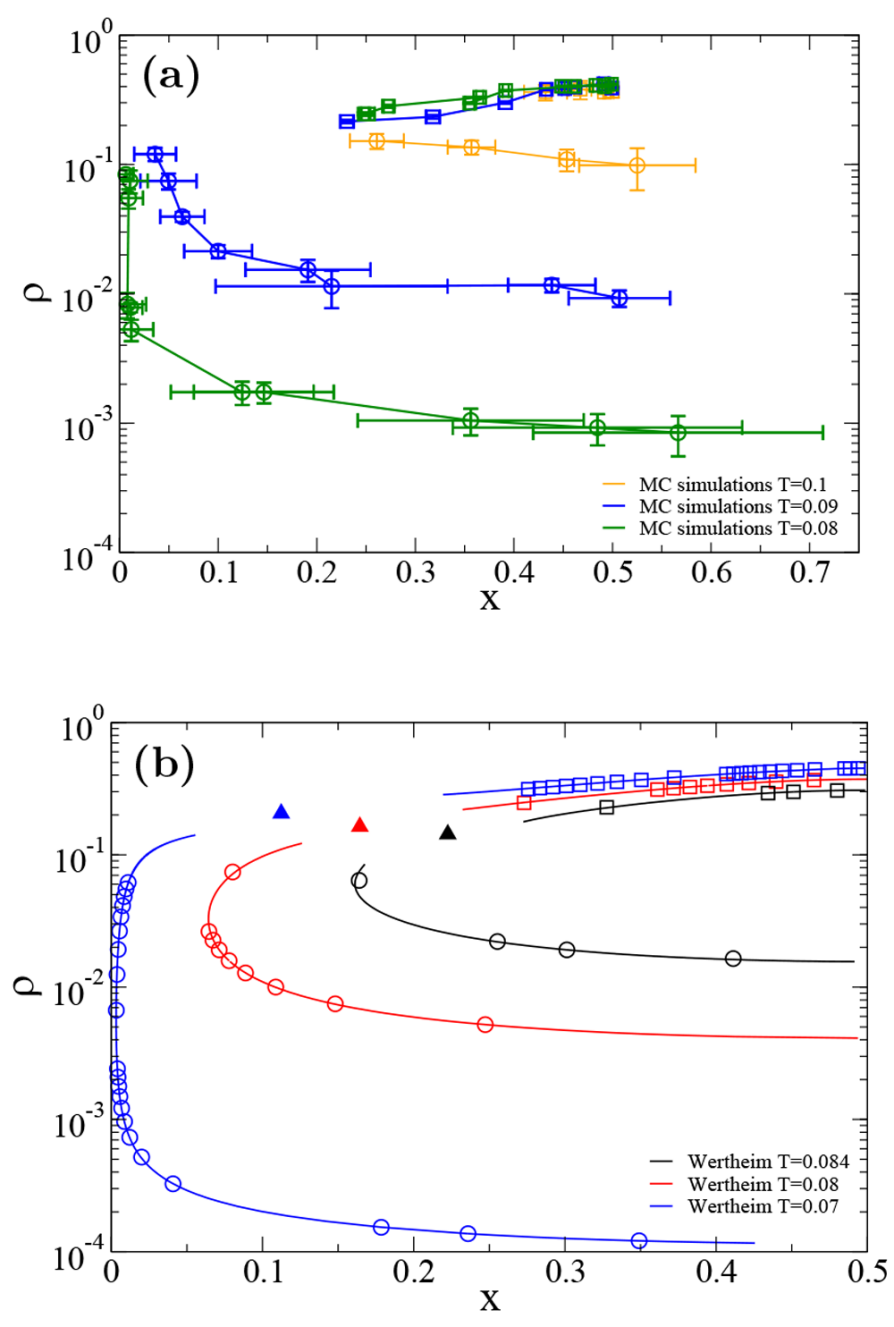
SAT-designed N2c8s2 binary mixture density-concentration phase diagrams for different temperatures. Comparison between the binodal curves obtained from Monte Carlo simulations (a) and the binodal curves computed within the Wertheim first order perturbation theory (b). Circles represent points belonging to the dew point curve, while squares represent points belonging to the bubble point curve. Triangles indicate critical points.

Going beyond Wertheim’s theory, we study the numerical phase behavior of the N2c8s2 mixture via Monte Carlo simulations in the Gibbs ensemble. Simulations are performed at different temperatures (*T* = 0.1, *T* = 0.09, *T* = 0.08) and, for each temperature, at different averaged (over the two boxes) densities and concentrations in order to compute the binodal curve in the density-concentration phase diagram, as shown in Figure 6a. System size is fixed at *N* = 500 particles for all simulations since equilibration of these systems at the (low) temperatures, where phase separation is located, is particularly challenging.^64^ This is reflected in the non-negligible error bars in Figure 6a; however, we argue that they are more significant than size-effect errors. Nevertheless, the trend of the numerical computed binodal curves as well as the topology of the density-concentration phase diagrams are the same as the Wertheim ones, as shown in Figure 6b. As commonly observed,^39^ Wertheim’s theory tends to overestimate the size of the coexistence region. In detail, the bubble point curves align almost perfectly, while Wertheim’s prediction places the dew point curves at densities higher than those of the simulated one. Still, Monte Carlo simulations confirm the phase diagram topology with the presence of an azeotrope at concentration 1/2 in the N2c8s2 binary mixture.

**Figure 7:**
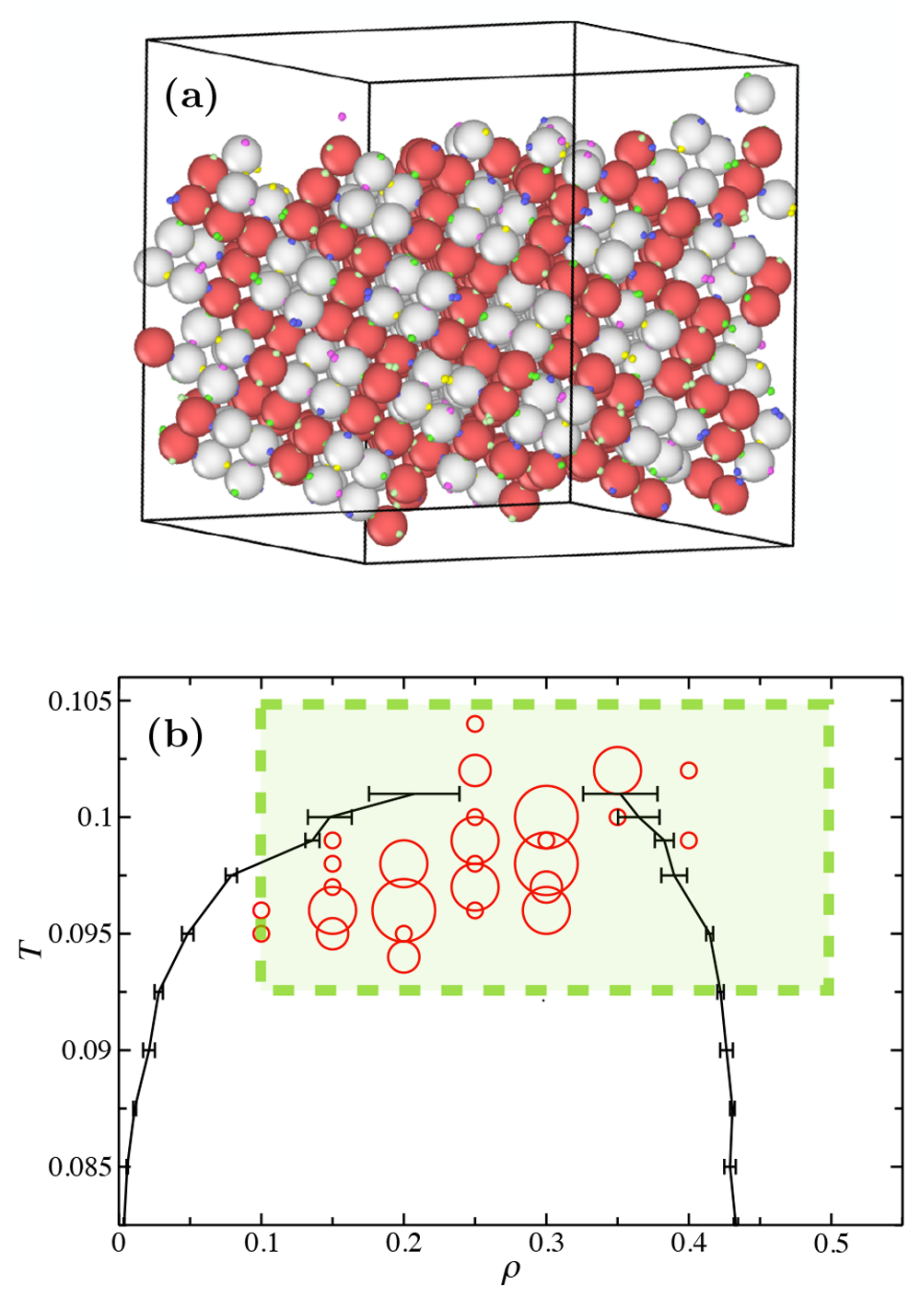
Nucleation plots. (a) Snapshot from a fully self-assembled solution prepared from a random configuration at *T* = 0.1, ρ = 0.3, and with patchy parameters fixed at θ_max_ = 0.98 and δ = 0.2. Patchy particles are colored red or white according to their species. (b) *T* – ρ phase diagram obtained from Gibbs ensemble simulations (black lines). The red circles are drawn in correspondence of the state points which nucleated. The radius of the red circles is proportional to the fraction of runs that successfully assembled within the simulation time of up to 5 × 10^8^ MC sweeps.

Next, we studied the self-assembly process through the azeotropic point. We prepare disordered configurations at equimolar concentration for different state points on a regular grid, with ρ ∈ [0.1, 0.5] and Δρ = 0.05, *T* ∈ [0.920, 0.104] and Δ*T* = 0.002. For each (ρ, *T*) state point we run 5 independent trajectories in the NVT ensemble with *AVB* biased moves^65^ (Monte Carlo Simulations: AVB Moves and Gibbs Ensemble section in the Methods). The considered state points are enclosed in the green shaded area in Figure 7b and each trajectory is run for 5 × 10^8^ MC sweeps or until crystallization. The centers of the red circles in Figure 7b represent the state points that crystallized within the simulation time. The diameter of each circle is proportional to the fraction of simulation runs (out of a total of 5 runs) that have crystallized at the corresponding state point. To understand why crystallization occurs only at selected state points, we superimpose (black line) the results from Gibbs Ensemble simulations that have been initialized at equimolar conditions. Error bars are computed on 10 independent runs for each temperature, and the black lines connecting the points are guides to the eyes to help identifying the gas and liquid branches. We confirmed that, once phase separation has occurred, both boxes (liquid and gas) are still found at equimolar concentration for all temperatures; i.e., we are always at azeotropic conditions. From Figure 7b it is clear that the self-assembly of the cubic diamond (red circles) occurs in correspondence with the phase separation boundaries. Self-assembly is aided by the formation of dense liquid regions during the phase-separation process. Interestingly, some state points in Figure 7b have nucleated outside the binodal boundaries but close to the critical temperature. The system thus represents an interesting example of nucleation aided by critical fluctuations, as predicted in ref (36) for isotropic interactions.

To summarize, the self-assembly pathway at the azeotropic point is the following: an equimolar disordered solution first generates equimolar critical fluctuations or first demixes in an equimolar dense liquid, which then crystallizes in an equimolar crystalline structure. Self-assembly under azeotropic conditions has the advantage of bypassing the difficulties associated with concentration fluctuations, which could otherwise severely limit the nucleation rate.

**Figure 8:**
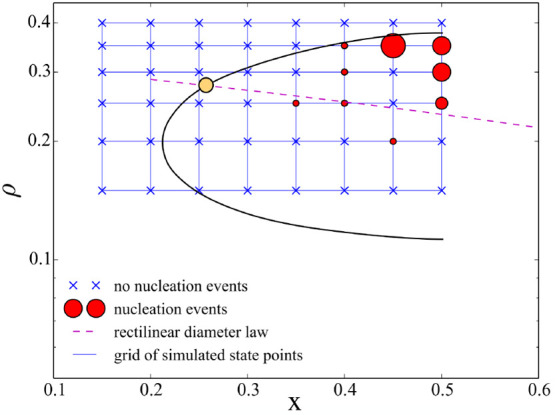
Nucleation events at *T* = 0.1 superimposed to a schematic representation of the density-concentration phase diagram. The binodal line is obtained from fitting the Gibbs ensemble results of Figure 6a. The formation of crystals with a fraction of particles in the cubic diamond phase equal or greater than 0.7 occurs mostly near the liquid branch around *x* = 0.5 (azeotropic condition). The blue grid defines all the state points considered; those showing no nucleation event are crossed out, while state points where at least one trajectory nucleated are represented with red circles. The radius of the circles is proportional to the fraction of trajectory that have nucleated within 3.5 × 10^8^ MC sweeps. The yellow circle indicates the critical point located at the intersection of the bindoal curve and the rectilinear diameter line, i.e., the (dashed) straight line passing through the midpoint of the tie lines connecting each pair of coexisting points.

**Figure 9:**
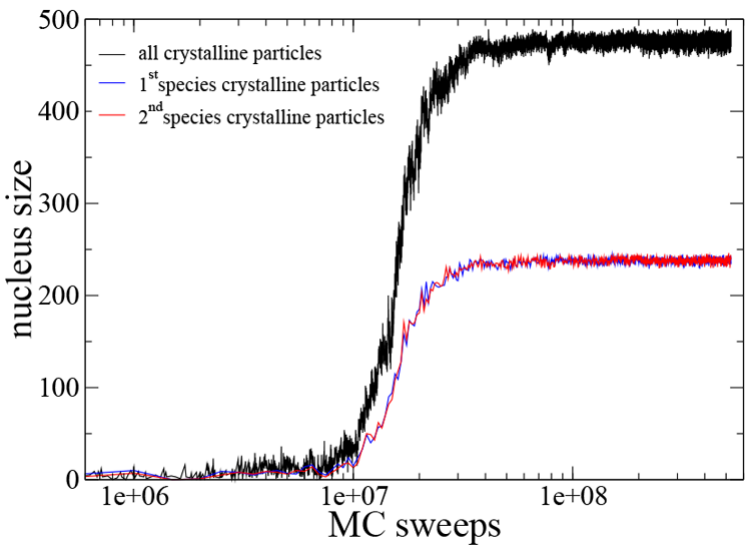
Progress of nucleus size for a nucleating trajectory at the azeotropic concentration *x* = 0.5, at temperature *T* = 0.1 and density ρ = 0.3. The blue and the red lines represent the number of particles in the crystalline phase of the first and the second species, respectively.

We further analyze the self-assembly process by studying the nucleation of solutions prepared at different densities and concentrations at temperature *T* = 0.1. The considered state points are located on the regular grids ρ ∈ [0.15, 0.4] and *x* ∈ [0.15, 0.5] with Δρ = 0.05 and Δ*x* = 0.05, as shown with blue lines in Figure 8. For each of these state points, we run 10 independent Monte Carlo simulations in the NVT ensemble with AVB dynamics and 500 patchy particles for 3.5 × 10^8^ MC sweeps. Running almost 500 nucleation trajectories, traditional molecular dynamics simulations are impractical for studying nucleation due to the slowness of particle diffusion. Since the kinetic contribution to the nucleation rate is usually negligible compared to the thermodynamic contribution (the nucleation barrier) we choose to employ biased MC simulations to explore the nucleation behavior across an extensive region of the system’s phase diagram. We look for state points exhibiting at least one nucleation event that gives rise to a cubic diamond with 350 or more patchy particles. In Figure 8 we mark these state points with red circles with a radius proportional to the fraction of trajectories that have nucleated. The fraction of particles in the cubic diamond phase are identified with local bond-order analysis.^66^ By superimposing the grid to the density concentration phase diagram, we can see that crystallization occurs exclusively within the liquid–vapor coexistence region. Figure 8 confirms that extended crystals are formed only close to the azeotropic point. Indeed it is exactly at azeotropic condition that the ratio between the two components in the liquid phase is the same as that of the cubic diamond crystal. This becomes evident upon examining Figure 9 which illustrates that at the end of the process almost all particles belong to the crystal phase, a consequence of the azeotropy of the liquid phase. Figure 9 also shows that the number of particles in the crystalline phase belonging to the first and second species is identical.

**Figure 10:**
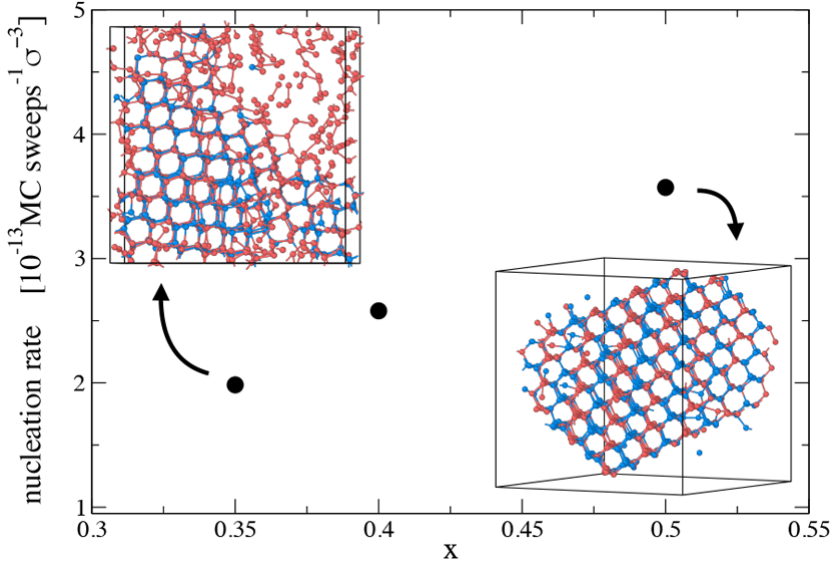
Nucleation rate and final configuration snapshots. Nucleation rate (black dots) as a function of concentration for systems of 1000 particles at temperature *T* = 0.097 and density ρ = 0.3. The two snapshots display the last configuration of a trajectory at *x* = 0.35 and at *x* = 0.5. Red and blue colors indicate the species to which a particle belongs: blue for the minority component, and red for the majority component.

In Figure 10 we show the nucleation rate computed, for each *x*, from 56 Monte Carlo trajectories ran at temperature *T* = 0.097, density ρ = 0.3 and with *N* = 1000 particles for three concentrations: *x* = 0.35, *x* = 0.4, and *x* = 0.5. The nucleation rate is estimated as the number of trajectory that successfully nucleates within 3.5 × 10^8^ MC sweeps, per unit of time and volume. Also at this temperature we observe that the nucleation rate increases toward the azeotropic concentration. The snapshots display the last configuration of a mixture prepared at the azeotropic condition (*x* = 0.5) and one away from it (*x* = 0.35). A visual inspection of these snapshots highlights that crystal growth is limited when the concentration of the liquid phase is different from the stoichiometric ratio of the target crystal components. Nucleation at the azeotropic point is advantageous as self-assembly can proceed up to 100% without one component depleting before the other and an extended crystal can form. On the contrary, at off-azeotropic conditions, the self-assembled cubic diamond coexists with a gas phase composed of the majority component that can only aggregate into chains. Going toward the azeotropic point, the density of the majority component diminishes until eventually all particles belong to the crystalline phase. Finally, regarding the quality of the crystals, we observe nuclei free from defects. The interaction matrix was indeed designed to avoid the hexagonal diamond phase and this also forbids the formation of stacking faults, which are the most common type of defects in cubic diamond crystals.

## Conclusions

Self-assembling complex structures requires designing complex interaction potentials that not only need to have the target structure as a free energy minimum but also have to avoid competing local minima that can kinetically frustrate the assembly process. In recent years it has become increasingly clear that using multicomponent mixtures can shift the problem from the need to accurately design the shape of the potential (e.g., introducing torsional interactions to assemble cubic diamond and avoid hexagonal diamond^23^) to the optimization of a generic interaction matrix between different components. This last problem is amenable to an effective numerical solution via the so-called SAT-assembly framework,^33^ where the interactions between the different components are found by solving satisfiability problems. But adding components increases the thermodynamic degrees of freedom, which considerably complicates the phase behavior and the assembly pathway.

In this work, we have shown that much of the thermodynamic difficulties can be removed by preparing the self-assembly pathway on an azeotropic point, where the system behaves effectively as a one-component mixture. We then show under which conditions we can include azeotropy in self-assembly designs.

As a proof of concept, we have focused on the case of patchy particles, which represent a convenient model for systems whose interactions can be described by isotropic repulsion and strong directional attractions. Exploiting the laws of mass-action, we have shown that in these systems azeotropy can be directly included in the interaction matrix. Different cases have been considered. The simplest condition, named *bond exclusivity*, asserts that an equimolar azeotropic point can be obtained by imposing that each patch has a unique interaction partner. The equimolar condition can be relaxed, and the azeotropic point can be located at a desired concentration vector **x**, by considering the *bond multiplicity* condition, which requires some patches to have more than one possible interaction partner. Finally, the *fully connected bond* condition, where each patch has one interaction partner on each of the species in the system, corresponds to a *always azeotropic* mixture.

We have then provided a fully worked example of a binary mixture designed to self-assemble colloidal diamond while avoiding the hexagonal form and that obeys the *bond exclusivity* condition. We have explicitly derived its phase diagram, both within Wertheim’s perturbation theory and via Gibbs ensemble simulations and have shown that it contains the predicted negative azeotrope at equimolar conditions. This class of phase diagram is characterized by a binary critical point line that approaches (*P*, *T*) → 0 as *x* → (0, 1), signifying that the system undergoes phase separation only during the process of mixing. Finally we have analyzed the self-assembly pathway for systems prepared at azeotropic conditions and shown that the pathway is the same as in one-component systems: more precisely, an equimolar mixture condensates into an equimolar liquid, which, given the coincidence in concentration between the crystal and the melt, then nucleates into a crystal that grows without concentration defects.

We believe that the ability to explicitly include azeotropic points into artificial designs represents an exciting step toward a fully consistent framework for the self-assembly of arbitrary structures. Efforts are now geared toward experimental realization of these designs, for example through wireframe DNA origami,^2,49,51,67^ that naturally encode binding specificity.

## Methods

### Patchy Particles

We consider multicomponent mixtures of patchy particles. Patchy particles are spherical colloids whose surface is decorated by attractive sites, named patches, and different species of patchy particles can differ by the number, the arrangement, and/or the type of the patches. To model their interaction we choose the Kern–Frenkel^68,69^ potential which describes hard-core spherical particles of diameter σ, interacting with an additional square well potential *V*_SW_ of depth ϵ and width δ, modulated by a term *F* depending on the patchy particles orientation. Two patchy particles attract in a strongly directional way if they are at a distance between σ and σ + δ. More precisely, the interaction potential *V* between particle *i* and *j*, with a center to center distance *r*_*ij*_ is
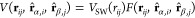
11where  indicates the position of patch α (β) of particle *i* (*j*), and
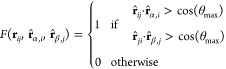
12

For identical patches, the Kern–Frenkel potential is characterized by the two independent parameters δ and θ_max_ that specify the range and the angular width of the patches respectively (see Figure 1) and that can be tuned giving rise to different phase diagrams.^59^

### DNA-Based Implementation

Patchy particle models are particularly suited to tackle the inverse self-assembly task since it is possible to control the valence and to encode the desired topology in the number, placement, and type of patches. Apart from their computational convenience, patchy particles are also experimentally viable systems: short ranged anisotropic interactions between colloidal particles have in fact been achieved via chemical patterning of their surfaces,^70−73^ and via modeling of their shape.^74^

The most promising approach to realize specific interactions uses DNA nanotechnologies to create a selective binding between particles: matchable colors^75^ correspond to complementary single DNA strands, equal colors to self-complementary sequences. Multiple color interactions can also be realized as discussed in Supporting Information V. Popular systems include DNA functionalized colloids^76^ or DNA origami^51,77−80^ where single strands of DNA are attached to well-defined positions on the particle surface.^77,79,81−84^

**Figure 11:**
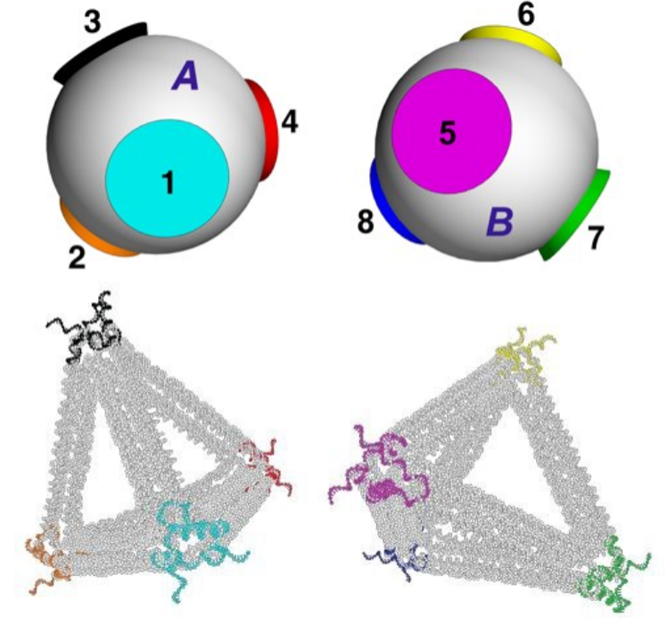
Sketch of two patchy particles realized through DNA origami. The four patches tetrahedrally arranged are mapped in single-stranded overhangs at each vertex of a tetrahedron made by nanoscale folding of DNA. Different interacting patches correspond to complementary single DNA strands and the self-interacting ones to palindromic DNA strands.

Figure 11 shows a possible realization of a binary mixture of patchy colloids with eight different patches (colors). The decorated hard-sphere colloidal model (which can be closely experimentally realized^85^) is displayed together with a DNA-origami implementation.^51^ The tetrahedron vertices are functionalized with DNA strands, exploiting DNA addressability to encode patch–patch interactions. In Supporting Information V we describe in full details an algorithm which allows us to determine the sequences of DNA strands that satisfy predefined bonding rules, applicable to both same-, distinct-, and multiple-color interactions. To apply the algorithm one needs to select the total length *n*_*s*_ of the oligomer grafted on each patch (for example an oligomer composed by six bases) and a rule quantifying the binding strength between any two oligomers (for example the melting temperature, estimated according to SantaLucia^86^ or the number of consecutive paired bases). See Supporting Information V for a full description of the algorithm.

### Wertheim Perturbation Theory

Here we report the results of the Wertheim first order perturbation theory^40^ that was originally developed to derive a mean-field theory of associating fluids and that can be easily generalized to patchy particles.^41,42^ Recently,^39,43−48^ the theory has been adopted to study in detail the static (e.g., percolation) and thermodynamic (e.g., phase behavior) properties of patchy particle systems, both in pure components and in mixtures, showing excellent qualitative agreement with numerical simulations. The main assumptions are that each attractive site cannot be engaged in more than one bond at the same time (one-bond-per-patch condition) and that a new bond occurs only between particles belonging to different clusters (loop formations are forbidden). Wertheim developed a perturbative method that, applied to patchy particles, estimates the effect of the attractive patches on the Helmholtz free energy of the reference system of hard spheres. The power of this theory is the chance to provide a good estimate of the Helmholtz free energy of a multicomponent system of patchy particles by only knowing the structure of the reference system and the interaction potential characterizing patchy particles. Here we follow the conventions of refs (44) and (47). The Helmholtz free energy per particle in units of *k*_B_*T* of a *n*-component mixture can be expressed as
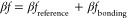
13

The reference free energy is the sum of the ideal gas contribution *βf*_ideal_ and of the hard spheres excess term *βf*_HS_. This hard spheres contribution takes into account the excluded volume of the patchy particles and it is given by the Carnahan–Starling formula^87^ since the different species have all the same diameter.
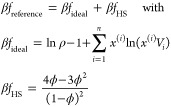
14where ρ is the density, *x*^(*i*)^ is the molar fraction of species *i*, *V*_*i*_ is the thermal volume, and ϕ is the packing fraction equal to ρ*V*_*s*_ where *V*_*s*_ = σ^3^π/6 is the volume of a single particle.

The bonding contribution contains the sum over the species (∑_*i*=1_^*n*^) and the sum over the patches of a certain species *i* (*∑*_α∈Γ(*i*)_); the number of patches of species *i* is denoted as *n*(Γ(*i*)).
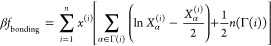
15*X*_α_^(*i*)^ is the probability that a patch α on a species *i* is not bonded and it is defined by the mass balance equation:
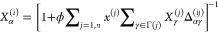
16where Δ_αγ_^(*ij*)^ does not depend on the species, since the diameter is always the same, and it is given by
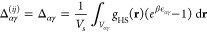
17where *g*_HS_ is the radial distribution function of hard spheres, *V*_αγ_ is the bonding volume and ϵ_αγ_ is the bonding energy both related to a bond between patches α and γ. As for any short-ranged patchy potential (in the single-bond per patch condition), the static properties are controlled by the bonding volume,^39^ i.e., the volume in which a particle can move while being bonded to another particle, which for the Kern–Frenkel potential assumes the following simple expression
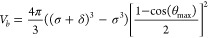
18

Δ_αγ_ characterizes the bond between the patch α on the patchy particle of species *i* and the patch β on the patchy particle of species *j*. Patches are in general different, and therefore, they can interact following different potentials (Kern–Frenkel in our case). In the following we consider that all bonds have the same bonding volume and we approximate the radial distribution function with an expansion around its value at contact, as detailed in refs (88) and (89). With these approximations, affecting the results only quantitatively, but not qualitatively, eq 17 becomes
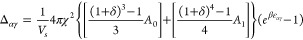
19with
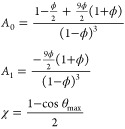
20

The theory allows for the computation of the Helmholtz free energy for any state point. Notice that solutions of the type *X*_α_^(*i*)^ = *X* in eq 15 (remembering that *∑*_*j*_*x*^(*j*)^ = 1) formally reduce the free energy of the mixture to that of a single component, i.e., the solutions correspond to azeotropic points.

### Isochoric Thermodynamics

One way to calculate the binodal curve for a single component system is offered by the integration of the Clausius–Clapeyron differential equation. Also in the case of multicomponent mixtures it is possible to define a set of differential equations that if integrated provides the binodal curve. Here we carry out the integration of these differential equations in the isochoric thermodynamics framework.^90,91^ We provide here a brief summary of this framework. In the canonical ensemble, the thermodynamic state of a *n*-component mixture is specified by temperature *T*, molar density ρ, and mole fractions *x*_*i*_. However, the mole fractions have some disadvantages: they are not independent variables and, conversely to density, they are dimensionless, causing the density mole fractions space to have an ill defined metric. On the contrary, in the isochoric thermodynamics the independent variables are molar densities ρ_*i*_ and the fundamental thermodynamic potential is the Helmholtz energy density Ψ. They are defined as
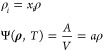
21where *A* is the Helmholtz energy and *a* is the molar Helmholtz energy, ρ is the molar density of the *n*-component mixture ρ = ∑_*i*=1_^*n*^ρ_*i*_ while **ρ** is the vector of molar densities **ρ** = (ρ_1_, ρ_2_, ..., ρ_*n*_).

The local curvature of the Helmholtz energy density is encoded in the Hessian matrix:
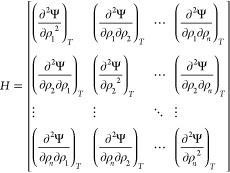
22

If it is positively defined, then the state is a stable state. We know that two phases (labeled ′ and ^*″*^ in the following) coexist in equilibrium at constant temperature if, along the phase boundary, the pressure and the chemical potentials of each component are equal for both phases. This means that the variation of the pressure and of the chemical potentials along the phase boundary must be the same for both phases:
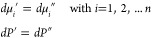
23having defined the chemical potentials and the pressure as μ_*i*_ = ∂Ψ/*∂ρ*_*i*_ and *P* = −Ψ + ∑_*i*=1_^*n*^ρ_*i*_μ_*i*_.

Integrating this system of first order differential equations allows us to numerically evaluate the coexistence region. For the isothermal phase equilibrium of a binary mixture we must solve:
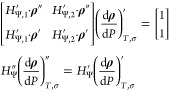
24where *H*_Ψ,*i*_ indicates the *i*-th row of the Hessian matrix with *n* = 2 in eq 22 and the subscript σ indicates that derivatives are calculated along the phase boundary.

By starting from available accurate initial values, the integration of the derivatives of the molar densities in the coexisting phases over the desired range of pressure predicts how molar densities of vapor and liquid change with pressure. This enables the construction of binary mixture pressure–concentration and density-concentration binodal curves. In summary, by knowing one pair of coexisting points, it is possible to determine the entire coexistence region by calculating how these coexisting points move along the binodal curve. Integration gets stiff and does not proceed further close to critical points, where the step-size of the adaptive step-size integrator^91^ progressively decreases as the Hessian determinant vanishes at the critical points. Hence critical points, indicated in Figure 3a by triangles, are computed by imposing the Hessian determinant to be zero and the stability conditions.

### Monte Carlo Simulations: AVB Moves and Gibbs Ensemble

When simulating patchy particle systems interacting via anisotropic and short-range interactions, rota-translation moves are not always sufficient to ensure a good sampling of the phase space. Indeed patchy particle self-assembly occurs when the thermal energy is much smaller than the bonding energy ϵ, which makes the Metropolis acceptance probability of a MC move that breaks a bond extremely low. Thus, almost all moves that try to break a bond are rejected not allowing the system to equilibrate. To overcome this drawback, we have introduced aggregation-volume-bias-moves (AVB)^64,65^ that facilitate bond breaking by enhancing the acceptance probability. In particular, there are two types of AVB moves: the AVB-B move and the AVB-U move. The AVB-B move attempts to create a bond by moving one patchy particle in the bonding volume (*V*_*b*_) of another patchy particle, thus giving rise to a bond between two patchy particles that were not bonded to each other. Conversely, the AVB-U move tries to break a bond by taking one bonded patchy particle outside the bonding volume (*V*_*o*_ = 4π*V* – *V*_*b*_) of the patchy particle to which it is bonded, thus eliminating an existing bond between a patchy particles pair. These moves are biased, and their acceptance probabilities are
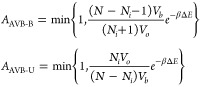
25where *N*_*i*_ is the number of particles that are bonded to particle *i*. Importantly, the acceptance probability of breaking a bond is enhanced with respect to the one of simple rototranslation move as the ratio *V*_*o*_/*V*_*b*_ is much larger than one since the bonding volume *V*_*b*_ is much smaller than its complementary volume *V*_*o*_ = 4*πV* – *V*_*b*_, where *V* is the volume of the simulation box.

In order to study the coexistence between two phases at a certain temperature, we employ Gibbs ensemble simulations,^92,93^ where coexistence occurs between two simulation boxes that virtually interact among each other without an explicit interface. In addition to rototranslational moves, the Gibbs ensemble incorporates volume moves (which alter the size of the two boxes, keeping the total volume fixed) and particle transfer moves (where a particle is moved from one simulation box to the other).

## Supporting Information Available

Explanation of azeotropy, example of a binary mixture satisfying the bond multiplicity condition, example of a binary mixture satisfying the fully connected bond condition, example of a 1:1:2 azeotropic ternary mixture self-assembling into a cubic diamond, description of an algorithm to generate DNA strands from the interaction matrix (PDF)
