# Supplementary material for: Engineering Azeotropy to Optimize the Self-Assembly of Colloidal Mixtures
Source: ACS Nano. 2023 Dec 4;17(24):24841–53. doi: 10.1021/acsnano.3c05569 (PMC10753881; doi:10.1021/acsnano.3c05569)
Supplement: Supplementary file 1 — nn3c05569_si_001.pdf [file nn3c05569_si_001.pdf]

Engineering azeotropy to optimize the self-assembly of colloidal mixtures

Camilla Beneduce[1], Francesco Sciortino[1], Petr Šulc[2,3], and John Russo[1]

[1]*Dipartimento di Fisica, Sapienza Università di Roma, P.le Aldo Moro 5, 00185 Rome, Italy*
[2]*School of Molecular Sciences and Center for Molecular Design and Biomimetics, The Biodesign Institute, Arizona State University, 1001 South McAllister Avenue, Tempe, Arizona 85281, USA*
[3]*School of Natural Sciences, Department of Bioscience, TU Munich, Am Coulombwall 4a, 85748, Garching, Germany*

Supplementary Materials

I Azeotropy

In systems of two or more components, both the pressure-concentration and the temperature-concentration phase diagrams exhibit a coexistence region. The presence of a coexistence region implies that the relative concentrations in the vapor and liquid phases are not the same. Supplementary Fig. 1 shows a qualitative pressure-concentration phase diagram for both an ideal (a) and a non ideal (b) binary mixture. In both cases, the coexistence pressures reduce to a single value when the first component concentration is equal to zero and to one, i.e. when the binary mixture becomes a one-component system. However, if the mixture strongly deviates from ideal behaviour, it can exist another point, named *azeotropic point* [1, 2], at concentration different from zero and one, where the coexistence region reduces to a single point. A multi-component mixture at the azeotropic point will separate into phases at the same azeotropic concentration, therefore behaving as a pure system.

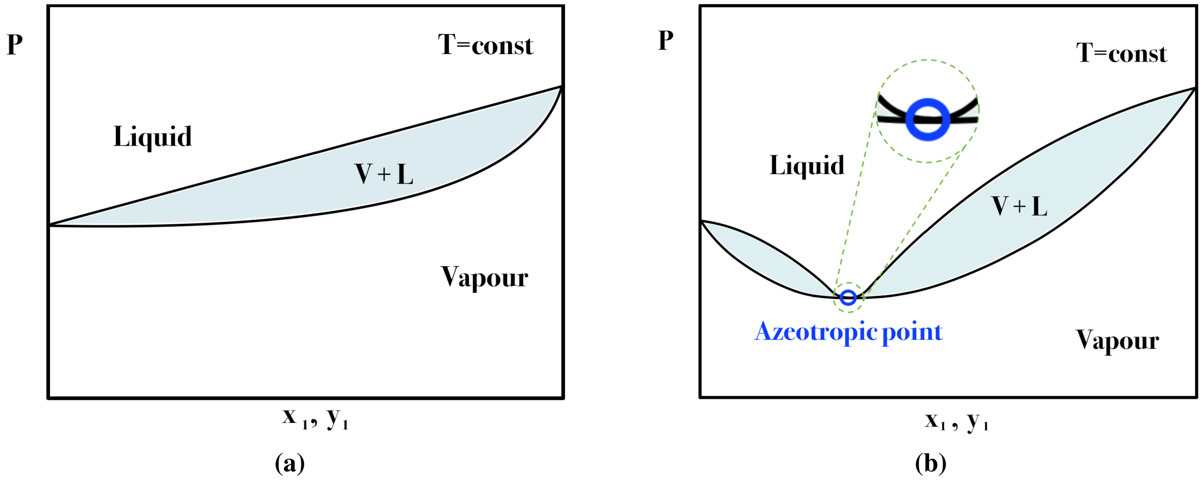

Supplementary Figure 1. Pressure-concentration phase diagram for an ideal binary mixture (a) and for a binary mixture with a negative azeotrope (b). The azeotropic point is located at the lowest pressure at which the liquid and the vapour phases can coexist at the specific temperature T. The inset highlights that at the azeotropic point the bubble point curve and the dew point curve are tangent; the bubble point curve is the locus of points where the first bubble of vapour appears when pressure is lowered starting from a point greater than the total vapour pressure, and the dew point curve is where the first liquid drop originates when pressure is increased starting from a point in the vapour phase.

In the context of distillation, the azeotrope is defined as the point where the fractional distillation process cannot modify the composition of the mixture since the distillate is again a

solution with the same composition as the starting one. Indeed, to obtain the distillate, the mixture in a liquid phase is heated until the bubble point curve is reached and then the vapour resulting from the phase separation is collected and condensed. Since phase separation occurring at the azeotrope preserves the ratio between components, the distillate will have the same starting azetropic concentration.

By preparing the mixture off the azetropic concentration, the fractional distillation process can still be exploited with some differences with respect to the ideal case where it allows to purify the more volatile component of the solution. In non ideal pressure-concentration phase diagrams, the azeotrope can be located at the lowest or at the highest pressure at which the liquid and the vapour phases can coexist at the specific temperature T. The first case is referred to as negative azeotrope and the second one as positive azeotrope. In a negative azeotropic mixture it is possible to purify either the more volatile component or the less volatile one depending if the system is prepared at a concentration smaller or greater than the azeotorpic one. Whereas a positive azetropic mixture cannot be purified from any of their components because fractional distillation eventually ends in the azeotrope. An example of a mixture having a negative azeotrope is the hydrochloric acid and water mixture, whereas for the positive case the most famous example is the ethanol–water mixture.

II 1:2 azeotropic mixture example

We analyse the example of a binary mixture of patchy particles, with four patches tetrahedrally arranged, whose ratio is $1:2$. An interaction matrix satisfying the bond multiplicity rules is:

$$\Upsilon_{\text{N2c6s3}} = \begin{pmatrix} 0 & 0 & 0 & 0 & 1 & 0 & 0 & 0 \\ 0 & 0 & 0 & 0 & 1 & 0 & 0 & 0 \\ 0 & 0 & 1 & 1 & 0 & 0 & 0 & 0 \\ 0 & 0 & 1 & 1 & 0 & 0 & 0 & 0 \\ 1 & 1 & 0 & 0 & 0 & 0 & 0 & 0 \\ 0 & 0 & 0 & 0 & 0 & 0 & 1 & 0 \\ 0 & 0 & 0 & 0 & 0 & 1 & 0 & 0 \\ 0 & 0 & 0 & 0 & 0 & 0 & 0 & 1 \end{pmatrix} \tag{1}$$

Notice that the naming convention of Υ_{N2c6s3}, which refers to the fact that there are two different species, six independent patches (colors) (six distinct rows or columns) and three self-interacting colors, is not unique, i.e. there are different ways of arranging 6 colors over two species. For the design in the matrix above, we look at the number and the placement of each 1 at each row. Patches under *bond exclusivity* constraint have a single one for each row, whereas patches obeying the *bond multiplicity* condition have two one for each row, as expected being the components ratio $1:2$, i.e. $n = 2$.

In this case, in the mass balance equations for $X_\alpha^{(i)}$ the sum over the species $(\sum_{j=1,2})$ still

drops out while the sum over the patches ($\sum_{\gamma\in\Gamma(j)}$) reduces to one or to two terms depending if patch α satisfies the *bond exclusivity* condition or the *bond multiplicity* condition, respectively. Together with the condition $\Delta_{\alpha\gamma} \equiv \Delta$ for all patches α and γ, this implies that the coefficient in front of $X_\alpha^{(i)}$ is the same for all species i. Patches belonging to the same species and characterised by the same row in the interaction matrix are equal and therefore they share the same probability $X_\alpha^{(i)}$. Hence, unlike the bond exclusivity case, not all patches are different and the number of distinct mass balance equations is smaller than $N_s \times N_p$. In our example, $X_1^{(1)} = X_2^{(1)}$ and $X_3^{(1)} = X_4^{(1)}$ and with simple algebraic steps we obtain the four following types of equations:

$$\begin{aligned}
&\text{if } \alpha = 1, 2 \\
&X_\alpha + 2\phi x^{(1)} {X_\alpha}^2 \Delta + \phi X_\alpha \Delta (x^{(2)} - 2x^{(1)}) - 1 = 0 \\
&\text{if } \alpha = 3, 4 \\
&X_\alpha + 2\phi x^{(1)} {X_\alpha}^2 \Delta - 1 = 0 \\
&\text{if } \alpha = 5 \\
&X_\alpha + \phi x^{(2)} {X_\alpha}^2 \Delta + \phi X_\alpha \Delta (2x^{(1)} - x^{(2)}) - 1 = 0 \\
&\text{if } \alpha = 6, 7, 8 \\
&X_\alpha + \phi x^{(2)} {X_\alpha}^2 \Delta - 1 = 0
\end{aligned} \tag{2}$$

We notice that if $x^{(2)} = 2x^{(1)}$ all the equations in Eq. 2 become equal to:

$$X + \frac{2}{3}\phi X^2 \Delta - 1 = 0 \tag{3}$$

indeed $x^{(1)} + x^{(2)} = 1$ and therefore $x^{(1)} = 1/3$ and $x^{(2)} = 2/3$. Hence the binary mixture displays an azeotrope behaving as a one component system at the specific non equimolar concentration of $x^{(1)} = 1/3$ and $x^{(2)} = 2/3$.

III fully-connected bond example

In the case of a binary mixture of patchy particles with four patches that differ only for their patch type, a possible interaction matrix satisfying the fully-connected bond recipe is:

$$\Upsilon_{\rm N2c6s2} = \begin{pmatrix} 0 & 1 & 0 & 0 & 1 & 0 & 0 & 0 \\ 1 & 0 & 0 & 0 & 0 & 1 & 0 & 0 \\ 0 & 0 & 0 & 1 & 0 & 0 & 1 & 0 \\ 0 & 0 & 1 & 0 & 0 & 0 & 0 & 1 \\ 1 & 0 & 0 & 0 & 0 & 1 & 0 & 0 \\ 0 & 1 & 0 & 0 & 1 & 0 & 0 & 0 \\ 0 & 0 & 1 & 0 & 0 & 0 & 1 & 0 \\ 0 & 0 & 0 & 1 & 0 & 0 & 0 & 1 \end{pmatrix} \tag{4}$$

Differently from the bond exclusivity interaction matrix that exhibits a single one for each row, this matrix has two ones for each row: the first is located among the first four columns (first species) and the other among the last four columns (second species). For a N_s-component mixture of patchy particles with N_p patches we will have a $N_s \times N_p$ matrix with N_s ones for each row: the first among the first group of N_p columns, the second among the second group of N_p columns, and so on.

In the following we demonstrate that with this binary mixture azeotropy is achieved without requiring equimolarity. Even better we show that this binary mixture exhibits azeotropy not only if the system is at a particular concentration, but whatever ratio the two species are mixed together.

In this case, in the mass balance equations for $X_\alpha^{(i)}$ the sum over the species $\sum_{j=1,2}$ does not drop out while the sum $\sum_{\gamma\in\Gamma(j)} X_\gamma^{(j)}\Delta_{\alpha\gamma}$ still reduces to one term as for the case where each patch can make a bond only with another patch. Indeed now, even if each patch makes a bond with two other patches, the patches involved in the bonds are located one on the first species and the other on the second species. Hence, for each patch α, $\Delta_{\alpha\gamma}$ is different from zero only for two patches, γ and δ, not belonging to the same patchy particle species. Therefore $X_\alpha^{(i)}$ is recasted as

$$X_\alpha^{(i)} = \frac{1}{1+\phi\left[x^{(i)}X_\gamma^{(i)}\Delta_{\alpha\gamma} + x^{(j)}X_\delta^{(j)}\Delta_{\alpha\delta}\right]} \tag{5}$$

Now we impose the equal bonding energy condition that allows to set $\Delta_{\alpha\gamma}$, for whatever α and γ, at the same value denoted as Δ. In this way, for each patch α, $X_\alpha^{(i)}$ becomes of the form

$$X_\alpha^{(i)} = \frac{1}{1+\phi\left[x^{(i)}X_\gamma^{(i)} + x^{(j)}X_\delta^{(j)}\right]\Delta} \tag{6}$$

In particular, considering the interaction matrix in Eq. 4, we have eight equations. For instance,

the ones for the patches 1 and 2 are:

$$
\begin{aligned}
X_1^{(1)} &= \frac{1}{1+\phi\left[x^{(1)}X_2^{(1)}+x^{(2)}X_5^{(2)}\right]\Delta} \\
X_2^{(1)} &= \frac{1}{1+\phi\left[x^{(1)}X_1^{(1)}+x^{(2)}X_6^{(2)}\right]\Delta}
\end{aligned} \tag{7}
$$

We notice that $X_1^{(1)} = X_6^{(2)} \equiv X$ and that $X_2^{(1)} = X_5^{(2)} \equiv X'$. This implies that

$$
\begin{aligned}
X &= \frac{1}{1+\phi[x^{(1)}+x^{(2)}]X'\Delta} = \frac{1}{1+\phi X'\Delta} \\
X' &= \frac{1}{1+\phi[x^{(1)}+x^{(2)}]X\Delta} = \frac{1}{1+\phi X\Delta}
\end{aligned} \tag{8}
$$

By replacing the expression for X' in the equation for X and vice-versa we obtain the two equal equations:

$$
\begin{aligned}
X + X^2\phi\Delta - 1 = 0 \\
X' + X'^2\phi\Delta - 1 = 0
\end{aligned} \tag{9}
$$

Therefore, satisfying the same equations, $X_1^{(1)} = X_6^{(2)} = X_2^{(1)} = X_5^{(2)}$. We are left to demonstrate that also $X_3^{(1)}, X_4^{(1)}, X_7^{(2)}, X_8^{(2)}$ are defined by equations equal to the ones in Eq. 9. Firstly we notice that if $X_7^{(2)} = X_8^{(2)}$ then $X_3^{(1)} = X_4^{(1)}$ and this would imply that $X_7^{(2)} = X_8^{(2)} = X_3^{(1)} = X_4^{(1)} \equiv X''$. Hence we can write

$$
X'' = \frac{1}{1+\phi[x^{(1)}+x^{(2)}]X''\Delta} = \frac{1}{1+\phi X''\Delta} \tag{10}
$$

which can be rewritten as

$$
X'' + X''^2\phi\Delta - 1 = 0 \tag{11}
$$

i.e. the same equation as the ones reported in Eq. 9. Therefore if $X_7^{(2)} = X_8^{(2)}$ then all the X_α, for whatever patch α, are equal. The equalities of all the X_α are valid for whatever value $x^{(1)}$ (and so $x^{(2)}$) takes. This means that this binary mixture is always an azeotropic binary mixture. Finally, the equality $X_7^{(2)} = X_8^{(2)}$ holds because, since the physics does not change if patch 7 is replaced by patch 8 and patch 3 is replaced by patch 4, then the equations must be invariant under these exchanges satisfying equalities $X_7^{(2)} = X_8^{(2)}$ and $X_3^{(1)} = X_4^{(1)}$.
In conclusion the bonding Helmholtz free energy is

$$
\begin{aligned}
\beta f_{bonding} =& x^{(1)}\left[4\left(\ln X - \frac{X}{2}\right) + \left(\frac{M}{2}\right)\right] + \\
&+ x^{(2)}\left[4\left(\ln X - \frac{X}{2}\right) + \left(\frac{M}{2}\right)\right] = \\
&= \left(x^{(1)} + x^{(2)}\right)\left[4\left(\ln X - \frac{X}{2}\right) + \left(\frac{M}{2}\right)\right] = \\
&= 4\left(\ln X - \frac{X}{2}\right) + \left(\frac{M}{2}\right)
\end{aligned}
\tag{12}
$$

where $n(\Gamma(i)) = M = 4$ (with $i = 1, 2$) since we deal with patchy particles species having both four patches. As expected, we notice that, for whatever concentration, $\beta f_{bonding}$ is equal to the free energy of a single component system.

IV 1:1:2 azeotropic ternary mixture self-assembling into a cubic diamond

In the main text we studied in detail the phase behaviour of the N2c8 solution for a facile self-assembly of the cubic diamond crystal.This mixture satisfies the bond-exclusivity condition and has an azeotrope at equimolar concentration, exactly at the same ratio at which the two species are present in the target unit cell. We demonstrated how this allows to overcome the compositional barrier, leading to a higher nucleation rate and the formation of larger crystals.
In this section we present an example of a mixture which obeys the bond-moltiplicity rule. We choose to design a three component system capable of spontaneously assembling into a cubic diamond at off-equimolar conditions. Our target structure is a 16 particle cubic diamond unit cell with 4 particles each of the first and second species, and 8 of the third species. We still use patchy particles with four patches tetrahedrally arranged, but now we move the azeotrope to a 1:1:2 ratio since we want it to be at the same concentration as the target structure. The bond-multiplicity rule must be satisfied: each patch making a bond with the majority component (the third species) must obey the bond exclusivity condition, whereas all other patches should have two bonding partners among patches of the first and second species. An interaction matrix guaranteeing the appearance of the azeotrope at $x^{(3)} = 2x^{(1)} = 2x^{(2)}$ is the following one.

$$
\begin{pmatrix}
0 & 0 & 0 & 0 & 0 & 0 & 0 & 0 & 0 & 0 & 1 & 0 \\
0 & 1 & 0 & 0 & 0 & 1 & 0 & 0 & 0 & 0 & 0 & 0 \\
0 & 0 & 0 & 0 & 0 & 0 & 0 & 0 & 0 & 1 & 0 & 0 \\
0 & 0 & 0 & 1 & 0 & 0 & 0 & 1 & 0 & 0 & 0 & 0 \\
0 & 0 & 0 & 0 & 0 & 0 & 0 & 0 & 0 & 1 & 0 & 0 \\
0 & 1 & 0 & 0 & 0 & 0 & 0 & 1 & 0 & 0 & 0 & 0 \\
0 & 0 & 0 & 0 & 0 & 0 & 0 & 0 & 0 & 0 & 1 & 0 \\
0 & 0 & 0 & 1 & 0 & 1 & 0 & 0 & 0 & 0 & 0 & 0 \\
0 & 0 & 0 & 0 & 0 & 0 & 0 & 0 & 1 & 0 & 0 & 0 \\
0 & 0 & 1 & 0 & 1 & 0 & 0 & 0 & 0 & 0 & 0 & 0 \\
1 & 0 & 0 & 0 & 0 & 0 & 1 & 0 & 0 & 0 & 0 & 0 \\
0 & 0 & 0 & 0 & 0 & 0 & 0 & 0 & 0 & 0 & 0 & 1
\end{pmatrix} \tag{13}
$$

The rows have either a single “one” in one of the last four columns, or they have two “ones” distributed among the first eight columns.

We found this interaction matrix within the SAT framework, where, in addition to the clauses ensuring the diamond structure topology, we added binary clauses to open the possibility of forming two bonds with the same patches while still maintaining bond exclusivity for all patches interacting with the third species.

To confirm that we have successfully designed a ternary mixture with an azeotrope at 1:1:2 composition, we run several Monte Carlo simulations with 500 particles for different temperatures and densities at the azeotropic concentration. In Supplementary Fig. 2(a) we show a snapshot of a typical configuration after the metastable liquid has formed but before nucleation. Since gas-liquid separation occurs at azeotropic conditions, the gas slab does not show excesses particles for any component. Supplementary Fig. 2(b) shows a snapshot of a correctly assembled cubic diamond crystal that formed at the target 1:1:2 composition. The last panel, Supplementary Fig. 2(c), shows the number of crystalline particles of each species as a function of Monte Carlo sweeps, demonstrating that the particles belonging to the first and second species have always the same concentration, which is half as that of the third species. We note also that the final nucleus comprises almost all particles, demonstrating that nucleation is occurring at azeotropic conditions.

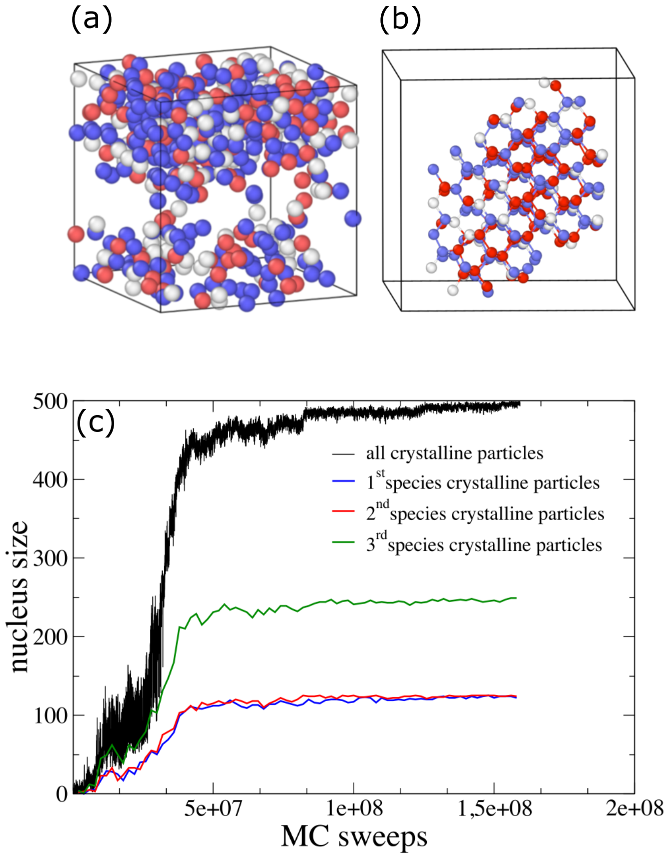

Supplementary Figure 2. Self-assembly of a cubic diamond from a ternary mixture prepared at the azeotropic concentration $\mathbf{x^{(3)} = 2x^{(1)} = 2x^{(2)}}$. (a) Coexistence between the gas and the metastable liquid phase. (b) Largest crystalline nucleus in the final MC configuration. (c) Size of the largest crystalline cluster as a function of MC sweeps (black line). The number of crystalline particles belonging to the third species (green line) is exactly double that of the first and the second species (blue and red lines, respectively) during the whole nucleation process.

To conclude, we designed a ternary mixture with an azeotropic point at the same concentration of the target crystal structure, and verified that it correctly assembles at these controlled conditions.

V An algorithm to generate DNA strands from the interaction matrix

In the article we introduced the interaction-matrix Υ, encoding the binding rules which must be satisfied by the patchy particles mixture. We also alluded to the possibility to use single-strand DNA sequences to encode the binding rules, exploiting either wireframe origami [3] or DNA-functionalized patchy colloids [4].

In this appendix we present an algorithm to select sequences of single strands of DNA to satisfy the desired interaction-matrix Υ. We remember that DNA is a sequence of four types of nucleobases: adenine A, guanine G, thymine T and cytosine C. The nucleobases can selectively bind to each other forming hydrogen bonds and the only possible base pairs are $A-T$ and $C-G$. We also recall that the melting temperature of a DNA oligomer is a function of the length of the DNA complementary sequence. For example, at a temperature at which DNA complementary sequence of length four are bonded, DNA complementary sequence of length two rarely bind.

We focus here on the case relevant for this article, but the method can be generalized to arbitrary binding rules. Specifically, we focus on a binary mixture of particles with four patches each (see Supplementary Fig. 3), interacting with the interaction matrix in Eq. 14 that satisfies the *fully-connected* bond condition.

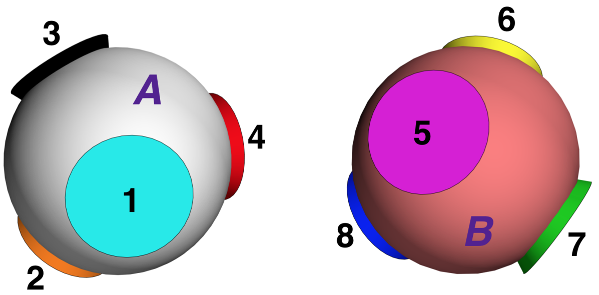

Supplementary Figure 3. 3D representation of patchy particles. A and B are two patchy particles species with four patches indicated by numbers. They interact establishing bonds through patches according to the interaction matrix in Eq. 14.

$$\begin{pmatrix} 0 & 1 & 0 & 0 & 1 & 0 & 0 & 0 \\ 1 & 0 & 0 & 0 & 0 & 1 & 0 & 0 \\ 0 & 0 & 0 & 1 & 0 & 0 & 1 & 0 \\ 0 & 0 & 1 & 0 & 0 & 0 & 0 & 1 \\ 1 & 0 & 0 & 0 & 0 & 1 & 0 & 0 \\ 0 & 1 & 0 & 0 & 1 & 0 & 0 & 0 \\ 0 & 0 & 1 & 0 & 0 & 0 & 1 & 0 \\ 0 & 0 & 0 & 1 & 0 & 0 & 0 & 1 \end{pmatrix} \tag{14}$$

The interaction matrix can also be represented as a list of nodes (the eight patches, labeled from 1 to 8 in Supplementary Fig. 3) connected by lines representing the 1s in the interaction matrix, resulting in the connected graph in Supplementary Fig. 4. We note that in the ”ring” forming graph (left graph in Supplementary Fig. 4), each patch binds to two different patches, while in the ”chain” graph (right graph), the first and the last node binds to one identical patches and to a different patch.

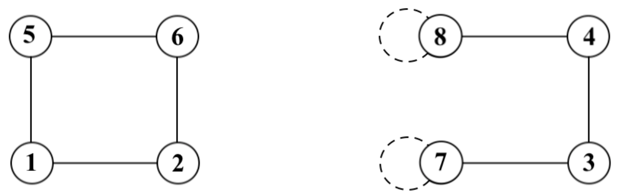

Supplementary Figure 4. Bond topology. The patch connections according to the color interaction matrix in Eq. 14 correspond to two disconnected bonded ”clusters”: a ring of four patches and a chain of four patches.

For this specific case, one need to find DNA strands that represent, as close as possible, the interaction matrix in Eq. 14 or equivalently the bond topology in Supplementary Fig. 4. Specifically, a strand must be able to form bonds with up to two different other strands. This ”double bonding” condition can be realized by defining a bond as a sequence of n_b consecutive base pairs (a realistic value could be $n_b = 4$) and a number of nucleotides in the DNA single strand n_s larger than n_b. An example of this type of double bonding, for $n_s = 6$, is shown in Supplementary Fig. 5.

To identify eight DNA single strand of length n_s which satisfy the interaction matrix in Eq. 14, we propose the following algorithm

- we generate all the 4^{n_s} different oligomers of length n_s and evaluate, for each pair of them the maximum number of consecutive bases n_{max} that bind to each other. It is important to remind that DNA has directionality and the two complementary strands that form it have opposite directions: one goes from the five-prime end to the three-prime end $5' \longrightarrow 3'$ and the other one from the three-prime end to the five-prime end $3' \longrightarrow 5'$. We then construct a $4^{n_s} \times 4^{n_s}$ matrix whose elements are the strength of the binding between the two strands. For clarity, we identify here the strength with the n_{max} value. A more elaborate formulation could use the binding Gibbs free energy or the strand pair melting temperature, calculated for example with the SantaLucia nearest-neighbor model [5].

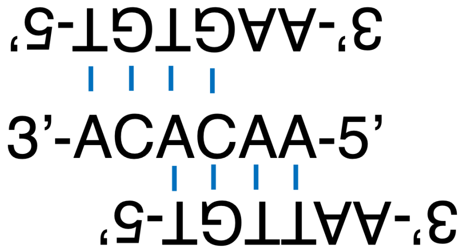

Supplementary Figure 5. Schematic example of the double bonding of a single DNA strand. Three single-strands DNA in which the central one is able to binds with four consecutive nucleotides with two other strands (reversed in their 3’-5’ order).

- Set a threshold $n_{\text{threshold}}$ for the largest value of $n_{\max}$ which can be safely assumed as non-bonding. In the case of $n_b = 4$, this can be chosen as 2. Indeed at the melting temperature of sequences of length 4, the binding probability of sequences of length 2 is negligible. Put zero in the $4^{n_s} \times 4^{n_s}$ matrix of $n_{\max}$ for all elements $n_{\max} \leq n_{\text{threshold}}$. In this way, the $4^{n_s} \times 4^{n_s}$ matrix has non-zero elements only for pair of sequences which bind to each other.

- Eliminate from the set of all possible strands (randomly) one of the two strands for which $n_{\max}$ is larger than n_b ($n_{\max} = 5$ and 6 in our example). This eliminates, among others, one of each pair of complementary strands and one of the two self-complementary sequences (palindromic in the DNA nomenclature). To eliminate the strand, it is sufficient to fill with zero the row and column associated to that strand. This makes sure that, among the remaining set of strands, the strongest binding is indeed the ones with $n_{max} = n_b$.

- By now, the $4^{n_s} \times 4^{n_s}$ matrix contains, weighted by their strength, all possible bonds between all possible strands, with values of $n_{\max}$ from $n_{\text{threshold}} + 1$ to n_b (from 3 to 4 in the specific case). This matrix can also be seen as a network of bonds (links) between the 4^{n_s} strands (nodes). We then run a search on this network to identify the desired bonding clusters. In the specific case (Supplementary Fig. 4) we first identify all rings of four nodes connected by links with $n_{\max} = n_b$. We then eliminate the rings which can be short-cut by a intra-ring bond and all rings in which one of the node is able to self-bind.

- For each of the remaining ring, we eliminate from the list of all possible DNA strands, the strands (i.e. the nodes) which are connected to the ring strands. This guarantee that the four strands defining the ring do not interact with any other remaining strand. Among the remaining nodes, we search for all chains of length four (in this specific case) on the network starting and ending with a node which is able to self-bind. As for the ring, we eliminate all chains in which intra-chain bonds are present.

- Iterating this procedure for each ring results in a list of DNA strands, all good candidates to experimentally realise the required interaction matrix. In a more refined treatment, each of these possible solutions can be examined to select the smallest variance in the ΔG of binding between different patches (compensating the different strength of the A-T and G-C pairing), and further restrict sequence selection so that ΔG distance between pairs with n_b and pairs with $n_{\text{threshold}}$ is as large as possible to minimize crosstalk. ΔG of binding between any pair of sequences would be calculated using SantaLucia's model, and can be obtained from with available strand analysis and optimization tools such as NUPACK [6].

In Eq. 15 we report one of the possible sets of 6 bases to originate 8 sequences that bind according to the request topology in Supplementary Fig. 4.

$$\begin{aligned}
① &= AAGGGG \\
② &= CCCCCC \\
③ &= ACACAA \\
④ &= AAGTGT \\
⑤ &= CCCTCA \\
⑥ &= AGGGGA \\
⑦ &= AATTGT \\
⑧ &= CACTAG
\end{aligned} \tag{15}$$

This sequence correspond to the following matrix of maximum number of paired base pairs.

$$\begin{pmatrix}
0 & 4 & 1 & 1 & 4 & 0 & 2 & 2 \\
4 & 0 & 0 & 1 & 0 & 4 & 1 & 1 \\
1 & 0 & 0 & 4 & 1 & 1 & 4 & 1 \\
1 & 1 & 4 & 1 & 2 & 1 & 2 & 4 \\
4 & 0 & 1 & 2 & 1 & 4 & 2 & 2 \\
0 & 4 & 1 & 1 & 4 & 0 & 1 & 2 \\
2 & 1 & 4 & 2 & 2 & 1 & 4 & 2 \\
2 & 1 & 1 & 4 & 2 & 2 & 2 & 4
\end{pmatrix} \tag{16}$$

Considering as bonded only the elements filled with a 4 (i.e. neglecting pairing of one or two nucleotides), this matrix coincides with the matrix in Eq. 14.

For the interested reader, we call attention on the fact that Eq. 14 has two identical row/columns. This makes it possible to reduce, if needed, the number of distinct colors from eight to six without altering the connectivity table. Strand ⑤ in Eq. 15 can be substituted with strand ② and strand ① can be substituted with strand ⑥ (or viceversa).

References

[1] Smith, J. M., Van Ness, H. C., Abbott, M. M. & Swihart, M. T. *Introduction to chemical engineering thermodynamics* (McGraw-Hill Singapore, 1949).

[2] Moore, W. J. *Physical Chemistry* (Prentice-Hall, 1962).

[3] Liu, W. *et al.* Diamond family of nanoparticle superlattices. *Science* **351**, 582–586 (2016).

[4] He, M. *et al.* Colloidal diamond. *Nature* **585**, 524–529 (2020).

[5] SantaLucia Jr, J. A unified view of polymer, dumbbell, and oligonucleotide dna nearest-neighbor thermodynamics. *PNAS* **95**, 1460–1465 (1998).

[6] Fornace, M. E. *et al.* Nupack: Analysis and design of nucleic acid structures, devices, and systems (2022).
